# Modelling the role of environmental circumscription in the evolution of inequality

**DOI:** 10.1098/rstb.2022.0291

**Published:** 2023-08-14

**Authors:** Cedric Perret, Thomas E. Currie

**Affiliations:** University of Exeter, University of Exeter, Penryn, Cornwall TR10 9FE, UK

**Keywords:** inequality, circumscription theory, migration, model, reproductive skew

## Abstract

Circumscription theory proposes that complex hierarchical societies emerged in areas surrounded by barriers to dispersal, e.g. mountains or seas. This theory has been widely influential but the lack of formal modelling has resulted in theoretical and empirical challenges. This theory shares parallels with reproductive skew models from evolutionary ecology where inequality depends on the capacity of subordinates to escape from despotic leaders. Building on these similarities, we extend reproductive skew models to simulate the concurrent evolution of inequality in many connected groups. Our results show that cost of migration does not directly limit inequality in the long term, but it does control the rate of increase in inequality. Second, we show that levels of inequality can be reduced if there are random errors made by dominants, as these lead to variations that propagate between polities. Third, our model clarifies the concept of circumscription by relating it to geographical features: the size of a region and the connectivity between polities. Overall, our model helps clarify some issues about how migration may affect inequality. We discuss our results in the light of anthropological and archaeological evidence and present the future extensions required to build towards a complete model of circumscription theory.

This article is part of the theme issue ‘Evolutionary ecology of inequality’.

## 1. Introduction

A major feature of the evolution of human societies has been the emergence of more complex, and unequal forms of organization [1]. Most hunter–gatherer societies and many small-scale horticultural societies are relatively egalitarian, with little differentiation in terms of status or wealth [2,3] and with leadership roles being limited, temporary, and/or based primarily on personal characteristics [4]. During the past 12 000 years, some societies saw political power become centralized and controlled by an elite few, who often had relatively more resources than the majority of the rest of the population. Understanding how and why such inequality has emerged and evolved are fundamental issues in the human sciences and have been heavily debated. Here, we draw on concepts from evolutionary ecology to develop a modelling approach to examine the ways in which the geographical distribution of resources and constraints on movement may affect the ability of emergent elites to control and extract resources from their population.

More than half a century ago, Robert Carneiro proposed a highly influential hypothesis [5], which argued that complex, unequal societies arise in *environmentally circumscribed* regions. These are areas in which there are limits to migration due to either geographical factors, e.g. narrow valleys surrounded by mountains, or social factors, e.g. densely populated areas with little free land close by in which to disperse. Carneiro pointed towards several examples where large-scale, complex societies arose in such places around the world including the ancient Egyptian state on the banks of the fertile Nile, where surrounding areas were desert, or in the mountain and coastal valleys of Peru. The basic reasoning is that some individuals (or groups) seek to control and subordinate others, and to extract resources from them in the form of tax or tribute. Potential subordinates facing this situation should seek to move away and avoid these costs but may be faced with barriers to migration that prevent this from happening or make it more difficult or costly.

Circumscription theory, as it is commonly referred to, has been attractive because it is built on a seemingly logical and compelling rationale, and seems to fit well with the observation that complex hierarchical societies often emerged in places where there were apparent barriers to dispersal and environmental heterogeneity [6–9]. But even discounting the fact that there may be other potential hypotheses that can explain socio-political evolution, the theory has been criticized on a number of fronts [10–13]. Some researchers have argued that there are real-world examples where land appears to be highly circumscribed but the societies there do not appear to be as strongly unequal or hierarchical as the theory would suggest. For instance, societies living in the densely populated and mountainous highlands of Papua New Guinea do not exhibit the kind of large-scale inequality with political organization beyond a local level (e.g. chiefdoms, or states) seen in many of the examples in which circumscription theory has been invoked (although there are systems of achievement-based hierarchy—so-called ‘Big Men’ systems) [14]. Furthermore, some authors argue that the emergence of the state did not necessarily follow the steps envisioned by circumscription theory. For example, some archaeologists argue that in the Valley of Oaxaca and lower Mesopotamia, the population actually declined before the rise of states [15].

Finding logical or empirical flaws in one part of such a theory does not necessarily mean that all parts are wrong, or that there are not useful insights that can be drawn from such attempts at theory building. Circumscription theory calls upon different elements including population pressure and warfare, as well as limits to migration. The primarily verbal basis of many debates means it is unclear how these elements may interact with other factors or other potentially important processes, or if all elements are necessary (see [6,16,17]). Circumscription is also a somewhat loose concept that has proven hard to define [12]. There may be several different factors that contribute to circumscription and affect the ease with which individuals are able to move away from potential dominants. These could include issues of connectivity (e.g. physical barriers that make movement difficult, or the existence of travel infrastructure such as roads), the amount of habitable land, or the costs of migration. The relative effect of these geographical factors is not clear; is a large but isolated island more circumscribed than a network of valleys?

Mathematical models of the evolution of inequality, including the role of circumscription, have been developed and can provide a means to assess the logical basis of theories and clarify how different processes may work [8,18–20]. Transactional models from evolutionary ecology, such as those found in biological reproductive skew models [21,22] and agent-based simulations of human societies [18], seem particularly relevant as they assess the relationship between migration and inequality. Transactional models abstractly conceive a group as comprising dominants and subordinates (i.e. they do not directly examine the process by which dominants obtain such a position; see [23]). The degree of inequality, or skew, is defined by the amount of resources the dominant is able to monopolize. Dominants want to extract as many resources as possible but may have to limit the degree of inequality to avoid the departure of the subordinates and subsequent loss of resource production. The incentive for subordinates to leave the group depends on the ease of migration and the options available to them outside the original group. Following this logic through, we can see that it holds similarities to key aspects of circumscription theory—higher inequality should be observed where there are barriers to migration or higher costs of moving to a new area.

The first aim of this paper is to clarify the role of circumscription in the capacity of dominants to impose inequality in human societies using reproductive skew modelling. To remain tractable, previous theoretical work made certain simplifying assumptions and there remain important gaps. First, the conditions under which barriers to dispersal would be expected to increase inequality are not clear. Indeed, the argument presented earlier relies on the assumption that out-migrating subordinates disperse to live alone, essentially giving up on living in groups [24]. Doing so, it overlooks the fact that individuals may have to disperse into existing groups that have their own degree of inequality and that outside options change with time. Changing the assumption from living alone to dispersing into another group seems to cancel the effect of greater costs of migration leading to greater inequality in some models [21], but not in others [18].

Second, reproductive skew models have tended to look only at the levels of inequality at equilibrium, after a long period of evolution. However, when considering the evolution of inequality we should consider that ‘circumscription’ may also affect the rate at which inequality emerges. Empirically, differences in dates of the emergence of complex, unequal societies that we see in the archaeological record of different places may be as much about how quickly inequality develops as they are about the ‘final’ level of inequality that a region exhibits. Third, typical reproductive skew models consider that dominants do not make mistakes when setting the level of inequality. However, bargaining games studying fairness have shown that errors can play an important role in shaping how an unequal distribution of resources can evolve [25,26]. Variations in the inequality set by dominants were also a key factor in the agent-based model by [18] mentioned above.

In order to address such issues, our model extends traditional reproductive skew models in three ways. First, we consider that subordinates migrate between groups (rather than dispersing to live alone), and we simulate the concurrent evolution of inequality in multiple groups. Second, we look at the whole process of evolution, which enables us to examine rates of change as well as long-term levels of inequality. Third, we consider that inequality can also vary because of errors made by dominants, or because of random events that make a dominant lose or gain power.

Another aim of our modelling approach is to disambiguate the role of different components of circumscription. Previous reproductive skew and related models focus on a generic cost of migration and consider an abstract setup with an infinite number of groups that are all connected. By contrast, discussions about circumscription in the anthropological and archaeological literature focus on geographical factors such as the size of a productive area or the connections between productive patches of land. In our model, we consider that the number of polities can vary. We also make the network structure explicit, meaning that polities can differ in their connectivity. Using this approach, we can disentangle the role of cost of migration, number of polities and connectivity with different network structures.

We emphasize that our aim in this paper is not to provide an empirical test of whether circumscription theory explains the emergence and evolution of inequality in human societies, nor even its relative importance compared to other potential mechanisms. Instead, our intention is to use this model to help guide theory development, to help clarify some of the issues raised above and to assess whether there are least some *plausible* mechanisms by which circumscription may shape inequality.

## 2. Method

### (a) Model description

We consider a population that is subdivided into a finite number, *P*, of polities. Polities are structured in a network, with a link between two polities representing that migration is possible between these polities. As we show later, we can configure networks so that their structure approximates the presence of the kinds of barriers to dispersal discussed in circumscription theory, e.g. mountains, seas, rivers, deserts. *P*_*i*_ refers to the set of neighbours of a polity.

Each polity contains a dominant and subordinates. We note that reproductive skew models were originally formulated to examine inequality within a group of individuals, and that for simplicity in formulating and discussing this model we also adopt this convention. However, the model is abstract enough that it could also represent differences between dominant and subordinate villages (as in seen in classical formulations of chiefdom forms of organization, or even larger polities, e.g. the centralized elites of a state and the rest of the population). Following previous models [18,22,27], a dominant can extract a proportion *z* of the resources of subordinates (we can think of this as some kind of tax or tribute on resources that is above those required by the population to meet minimum survival and reproduction needs). Any value *z* > 0 represents some degree of inequality. If *z* = 0 then subordinates keep all their resources, whereas if *z* = 1 then the dominant takes all of the extra resources. The level of inequality is set by the dominant, but subordinates can respond by migrating away from the group. Following reproductive skew theory, we consider that subordinates use migration as a threat, and that the dominant increases inequality up to the limit at which subordinates would leave. However, migration does not happen or is at least not explicitly modelled.

Subordinates compare their expected payoffs if they migrate and if they do not. Subordinates will migrate if

2.1 $$(1-z_{i})b_{i}<(1-c_{m})\max_{\left\{j\in P_{i}\right\}}(1-z_{j})b_{j}.$$

The left part of equation (2.1) represents the payoffs that subordinates will obtain if they stay in the same patch *i* with the given inequality *z*_*i*_ and resources produced *b*_*i*_. The right part represents the payoffs that subordinates will obtain if they migrate to the best possible patch among their neighbours *P*_*i*_. It is the resources they would obtain in the patch discounted by a cost of migration *c*_*m*_. We consider here that polities and patches do not vary in their production of resources, and we drop *b* from subsequent equations.

The dominant calculates the share of resources that they need to concede to their subordinates. To prevent subordinates from migrating the dominant will need to match the lowest level of inequality found in neighbouring polities, while also factoring in the cost of migration. The value of inequality at the next time step for a given polity *i* can be found by solving this equation for *z*_*i*_:

2.2 $$z_{i}(t+1)=1-(1-c_{m})\max_{\left\{j\in P_{i}\right\}}(1-z_{\;j}).$$

It is equal to the share of resources received by the ‘richest’ subordinates of neighbouring polities $\max_{\left\{j\in P_{i}\right\}}(1-z_{\;j})$, discounted by the cost for subordinates to migrate to this polity (1 − *c*_*m*_). Unlike traditional reproductive skew models, the payoff for subordinates migrating is not a constant value but depends on the inequality in other patches. Thus, we use simulations to model the evolution of inequality. At each time step, one random group is chosen and the dominant of this polity updates the level of inequality to *z*_*i*_(*t* + 1).

Under the default assumptions the dominants will always set the optimal level of inequality for themselves, i.e. the level that is as high as possible without providing an incentive for subordinates to migrate. However, inequality at the next time step can differ from the optimal inequality for several reasons. First, in the real world, dominants and subordinates have limited cognitive capacities and information, and they cannot calculate the optimal level of inequality perfectly. Second, inequality also depends on the balance of power between dominants and subordinates. For instance, inequality can vary when dominants gain or lose wealth, or when the dominant gets replaced by revolution, or when power gets transferred to their offspring [28–30]. To capture this uncertainty or instability, at each time step the inequality is increased or decreased by a random value drawn from a uniform distribution [−ϵ,+ϵ ] with a probability *μ*_r_. A low value of ϵ approximates situations where a leader’s position is fairly stable, e.g. supported by institutions and with clear heritability rules, where mistakes in setting the level of inequality may be small. A high value of ϵ approximates situations where leadership is based on achievement and inequality can vary depending of the actions of the leader, or when the leader dies without clear rules of succession.

### (b) Analysis

We are interested primarily in the rise of inequality from an egalitarian starting point. Therefore, the initial level of inequality in the model is set at a low level and randomly drawn from a uniform distribution defined on [0.05, 0.10]. One time step is defined as *P* update, that is on average, each polity has updated its inequality once.

The analysis is divided into two sections. First, we investigate when and how limits to migration affect inequality. The goal here is to describe how migration between groups and instability affect the dynamics of the model and how the features of this model may lead to results that differ from traditional reproductive skew models. To do so, we start from a model that is the closest to classic reproductive skew models, i.e. all groups are connected and there is a large number of groups. We also consider the same abstract definition of cost of migration, that is the cost of leaving a group and moving elsewhere.

In the second section, we move towards a more realistic instantiation of the model, where we investigate the role of geographical circumscription. To do so, we differentiate cost of migration (i.e. the cost of changing groups) from connectivity (the number of groups that can be reached) and size of the area (i.e. the total number of polities existing, in whatever way they are connected).

## 3. Results

### (a) Investigating the role of cost of migration in the evolution of inequality

**Figure 1.**
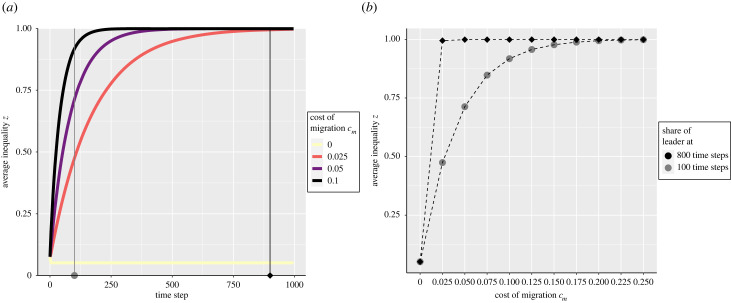
(*a*) Average inequality as a function of time and cost of migration *c*_*m*_. (*b*) Average inequality as a function of cost of migration *c*_*m*_ after 100 time steps and 900 time steps. The inequality presented is the average inequality across 100 replicates. The parameters used are *P* = 49, all polities are connected, *μ*_*r*_ = 0.01, ϵ=0.

Figure 1 shows how inequality changes with time as a function of different costs of migration. First, it shows that when there is no cost of migration *c*_*m*_ = 0, inequality initially reduces to its minimum possible level. This is the expected result because the dominant of a polity next to a polity with lower inequality has to reduce its inequality to avoid departure of its own subordinates. Once all groups are at the lowest value of inequality, they are all equally attractive to subordinates and inequality remains stable. However, figure 1 shows that when there is a cost of migration, all polities eventually end up with a very high level of inequality. This is the case even under a very low cost of migration $c_{m}=2.5\%$. While this may appear surprising, it can be explained by the fact that when a dominant updates its inequality, the share that the leader needs to concede is the share that the leader of the most equal polity provides to their followers, discounted by the cost of migration, 1 − *c*_*m*_. Note that for any non-null cost of migration, (1 − *c*_*m*_) < 1 and thus, the new inequality will always be slightly higher than the inequality of the other patch. As each polity repeats this process, inequality increases to the maximum value possible, *z* ≈ 1. Figure 1*b* shows that what the cost of migration does affect is the rate of increase in inequality. This is important because our results show that the process of increase of inequality can be slow, for instance it takes almost 1000 updates for each dominant to reach the maximum level of inequality at a low cost of migration, $c_{m}=2.5\%$. Applying this insight to the archaeological record, this result is consistent with the idea that regions that had higher costs of migration would show evidence of large differences in inequality at a relatively earlier period in time than regions that had lower costs (see figure 1*b*).

**Figure 2.**
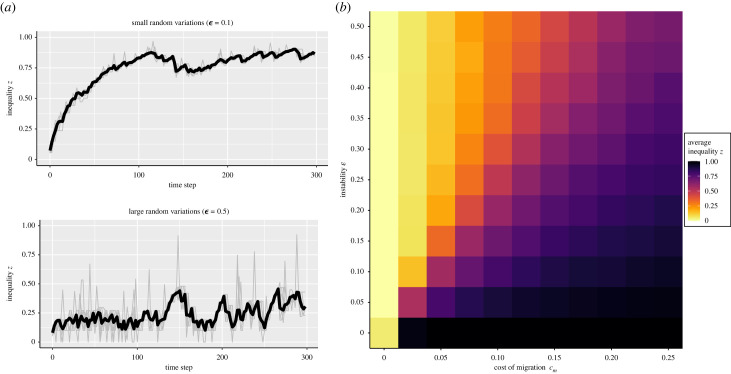
(*a*) Inequality for each polity (in grey) and average inequality (in black) for a single run considering small or high random variations for 49 polities. (*b*) Average inequality as a function of cost of migration *c*_*m*_ and instability ϵ. The inequality presented is the average inequality across 100 replicates, and on the last 500 updates after 29500 updates. The parameters used are *μ*_*r*_ = 0.01, *d*_max_ = 100 (all patches are connected).

We now consider the evolution of inequality in the presence of random variation due to errors or other processes. Figure 2 shows that the presence of random shocks makes inequality within patches fluctuate either up or down, but leads on average to a lower inequality at equilibrium. Note that this result holds even though there is the same probability for the random variation to increase or decrease inequality. This is because a given group will increase its inequality only if *all* its neighbours have increased their inequality, while it only needs *one* of its neighbours to decrease its inequality to cause the group to decrease its inequality. This effect is also visible in the left part of the figure, where there are often short spikes of polities with higher inequality than average (grey peaks). However, as other groups do not respond by increasing their inequality the original polity has to reduce its level of inequality at the next time step to avoid subordinates leaving.

When cost of migration is greater than zero, the average level of inequality is a balance between cost of migration pushing inequality up, and random variation pushing it down. Cost of migration still plays an important role in the presence of random variation because it controls the rate of increase of inequality. This affects both the point at which inequality begins to rise appreciably, and also enables inequality to ‘recover’ from reductions in inequality.

### (b) The role of geographical circumscription on the evolution of inequality

In the previous section, we explored the dynamics of the model and investigated the effect of cost of migration on inequality. We now look at the effect of different components of circumscription on inequality. To vary connectivity, we first consider that polities are organized on a square grid with a distance of 1 between two adjacent squares. Connections exist between two polities if the distance is less than a maximum travelling distance *d*_max_. We then move to heterogeneous networks to capture more realistic scenarios. In the following results the cost of migration (*c*_*m*_), and the parameters relating to instability (*μ*_*r*_, ϵ) are kept constant.

**Figure 3.**
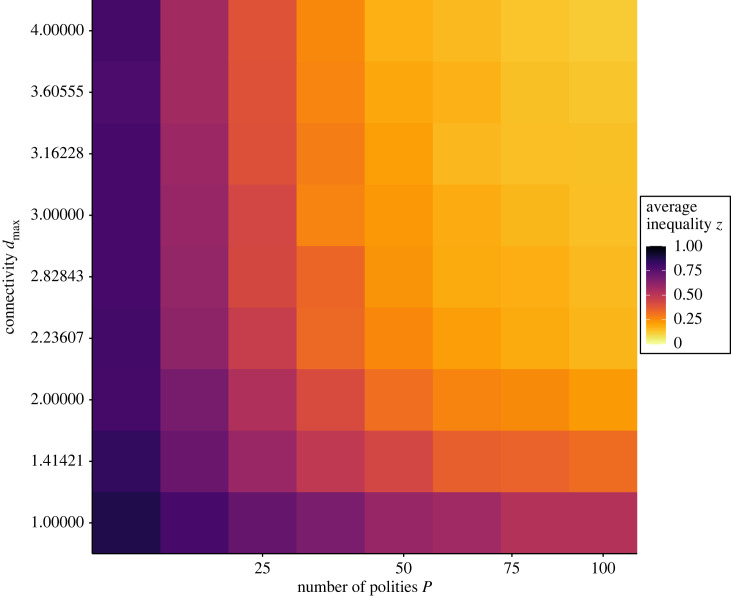
Average inequality as a function of number of polities *P* and connectivity, controlled by maximum travelling distance *d*_max_. The inequality presented is the average inequality across 100 replicates and on the last 500 updates after 29 500 updates. The parameters used are *μ*_*r*_ = 0.01, ϵ=0.25, *c*_*m*_ = 0.05. The network structure is not periodic, which means that polities on the edge have fewer neighbours. Electronic supplementary material, figure S2 shows that considering periodicity does not qualitatively change the results.

Figure 3 shows that decreasing the number of polities and decreasing the connectivity between polities both lead to increases in inequality. In other words, smaller and less connected areas exhibit more inequality. These effects are a result of the generation of variation, or fluctuations, in inequality within polities and the subsequent dynamics of changes that these fluctuations induce in connected polities. In the absence of any random variations (ϵ = 0), increasing or decreasing connectivity or the number of polities do not affect inequality either in the short or long term (electronic supplementary material, figure S1). However, if we allow for random variations to occur (ϵ>0) then increasing the number of polities increases the probability that in a given time step, a leader of a polity will randomly reduce its inequality (electronic supplementary material, figure S3). Such reductions in inequality will then cause connected polities to reduce their inequality to avoid a loss of subordinates, as we saw in the initial results. Connectivity then controls how easily a decrease in inequality in a polity will propagate in the landscape of polities. When polities are not well connected then even if a dominant in one polity does propose a lower level of inequality, it is more difficult for that value to spread. In the presence of a large number of highly connected polities, there will always be a leader proposing a better deal to subordinates of other polities.

Various other interesting effects can be seen when looking more closely. First, figure 3 shows that the effects of increasing connectivity and increasing number of polities are nonlinear. For scenarios with low connectivity and a small area (the bottom left region of the figure), small changes in either parameter have a large effect on inequality. However, in scenarios with a large area and well-connected polities (upper right region of the figure), small changes have a very limited effect. In other words, a circumscribed area is one that is both fairly small and has low levels of connectivity. Second, an increase in number of polities, which can be seen by looking across figure 3 horizontally, leads to decreases in inequality, even when connectivity is low. This means that two polities that are connected to the same number of other polities could still show important differences in inequality depending on how many polities there are in total. It highlights that it is not only the number of direct neighbours that a polity has that matters, but rather the whole landscape is important. Third, the effect of connectivity, which can be seen by looking at figure 3 vertically, depends on the number of polities. It has less of an effect when the number of polities is low, and a higher effect when the number of polities is high.

### (c) Circumscription in more realistic scenarios

So far, we have considered basic scenarios where all polities have the same number of neighbours. Here, we look at more realistic scenarios to see how network structure affects inequality.

**Figure 4.**
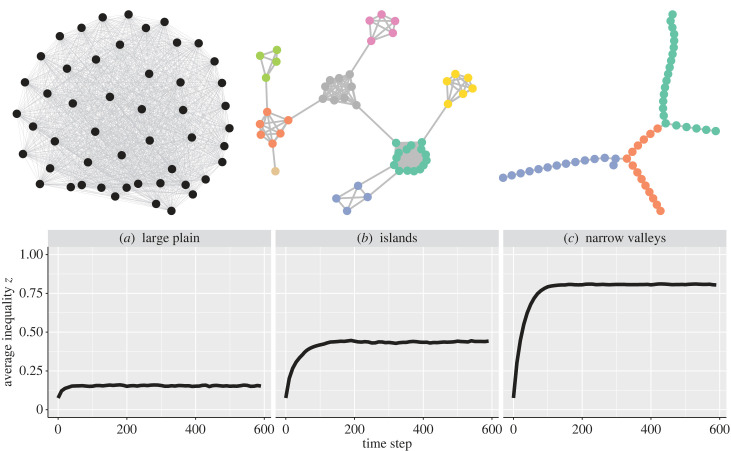
Evolution of inequality (averaged across 500 replicates) as a function of time for different scenarios: (*a*) a single plain where all polities are connected, (*b*) islands where polities within a island are all connected and (*c*) valleys where polities are organized in stepping stone manner. Examples of such networks are depicted above the graphs. The parameters used are *P* = 50, *μ*_*r*_ =0.01, ϵ=0.25, *c_m_* = 0.05.

We compare three scenarios that are often cited as examples in discussing circumscribed environments: (1) a population of polities spread on a large plain, (2) more island-like situations or (3) narrows valleys. The number of polities remains the same but there are differences in connectivity. In the large plain, all polities are connected. In the islands scenario, all polities on an island are closely connected, with fewer connections to other islands. In the narrow valleys scenario, all polities within a valley are connected in a stepping-stone fashion (one after each other) and there is only a single link between different valleys. Note that although we label the second scenario as ‘island’, this kind of situation could represent more that just physical islands in bodies of water, but rather any situation where there are pockets of productive land surrounded by areas that are less productive (e.g. areas of alluvial soils separated by heavily forested land with poorer soils), or where there are barriers to dispersal (e.g. larger valleys in a mountainous regions). Examples of these networks are shown in the top part of figure 4.

To build the network structures of islands and valleys scenarios, polities are randomly assigned to a number of cliques (10 for islands and 3 for valleys). For each network type the numbers of polities and cliques are constant, but the number of polities within a clique can differ. Within each clique, the polities are either connected to each other in the island scenario, or connected following a stepping stone network in the valleys scenario. One link is added between a random polity of each clique. Note that this generative model is similar to the stochastic block model [31]. The main difference is that we control the number of links between or within a clique, while the stochastic block model algorithm uses probabilities.

The bottom part of figure 4 confirms previous intuitions that islands lead to higher levels of inequality than a plain, and narrow valleys result in an even higher inequality than islands. This is because in the island scenario, if islands are large enough, multiple polities are connected to each other and any reductions in inequality can spread fairly easily to the other polities on the island. However, in the narrow valleys scenario, even polities within a valley are poorly connected and behave as isolated polities. Electronic supplementary material, figure S4 shows that the level of inequality in an island scenario strongly depends on the size of the island. However, the size of a valley and the number of polities in a narrow valley scenario have no effect.

## 4. Discussion

In this paper, we have explored how limits on migration may affect the evolution of inequality in human societies. Inspired by aspects of circumscription theory, we have adapted reproductive skew models to examine how geographical factors may affect what share of resources a dominant individual can extract from subordinates. Our results indicate that increasing costs of migration limit the outside options for subordinates and can lead to inequality evolving at a faster rate. In considering how different groups are connected in a landscape, we show that having fewer groups and/or lower levels of connectivity between groups leads to higher levels of inequality. Running the model on networks that approximate different geographical scenarios we find support for the idea that inequality tends to be lower when there are larger open plains than when groups are more fragmented or isolated with relatively few links to other groups. Overall, our model helps clarify some issues about how migration and the geographical distribution of resources may affect inequality. We discuss these findings in relation to real-world anthropological and archaeological information and suggest ways in which this modelling approach could be extended in future.

First, our analysis of the cost of migration reveals an interesting distinction between the overall level of inequality that is reached and the rate at which inequality evolves. We find that varying the cost of migration does not itself cause variation in the level of inequality in the landscape in the long run. In fact, under all cost of migration parameter values the maximum value of inequality is eventually reached. What altering the cost of migration does do is to affect the rate of evolution of inequality—when costs are higher, moving away from more despotic leaders is more difficult, and therefore it takes less time for dominants to impose greater inequality. The circumscription hypothesis is often framed as explaining why hierarchical, unequal forms of organization like the state arose first in some places but not others [5] (and clarified again in [16]). However, our results draw attention to the effect of limits to migration as a more general process that may affect the rate at which inequality develops. Quantitative comparative analyses of historical and archaeological time series data in different environments could potentially assess the role of circumscription in the rates of evolution of inequality [29,32].

We should emphasize that under the assumptions of our model our finding that the maximum level of inequality is always reached occurs in the absence of other processes. Indeed, once fluctuations and instability in the level of inequality that individual dominants propose are introduced, the average level of inequality that is achieved is limited. In the real world it is likely that other mechanisms, which we have not included here, would also limit inequality. For instance, subordinates can also build levelling coalitions to remove dominants that are being too despotic [33–35].

The results from our model highlight the importance of considering how inequality can be affected not only by the internal dynamics within a single group but also by what is happening in the ‘landscape’ of other groups to which it is connected. Inequality within a polity may reduce due to mistakes by the leader in setting the level of inequality, or because a leader suffers a revolution and loses power. This means that other polities have to align, which on average reduces inequality across the landscape. These fluctuations in the level of inequality proposed by leaders creates a market that forces other leaders to propose a better ‘deal’. Importantly, we have shown the counterintuitive result that this effect happens even if the random variations are equally likely to increase or decrease inequality within a particular polity. This effect of random variations is in line with the economic literature on bargaining games, such as ultimatum games. Similar to our results, models and laboratory experiments of such games have shown that unfairness usually evolves, but that uncertainty and errors will limit unfair offers [25,26,36]. The effect of this variation in proposals by dominants revealed in our model helps to explain some seemingly conflicting results in the previous literature. Reproductive skew models that have investigated migration between groups (rather than subordinates dispersing to live alone [21]) also find that the maximum level of inequality is reached. However, an agent-based model, which addressed different but related issues, found that the level of inequality was actually affected by cost of migration [18]. This difference is due to the fact that the reproductive skew model assumed that all individuals follow the optimal strategy without error, while the agent-based model considered that dominants sometimes follow a non-optimal strategy.

This view of inequality being set by the ‘market’ of dominants and subordinates has already been well discussed in literature on partner choice [37,38] and it aligns well with previous models by [19,27], which modelled the emergence of leadership and inequality. In the latter models, leaders extract a cost from subordinates but play a functional role by punishing free-riding in the group, thus enabling the production of public goods. They consider that subordinates can replace the leader (at no cost) for another individual within the group who is ready to lead for a different price. This results in a very low level of inequality, given that subordinates are efficient in choosing their new leaders. In our model, subordinates cannot easily overthrow their leader but need to migrate to change leader. This means that the number of other potential leaders is limited and the outside opportunities available in other groups depend of the landscape. Therefore, in our model, we have explicitly connected the kind of geographical features described in circumscription theory with the market of dominants and subordinates, i.e. outside opportunities depend on the degree of isolation, and the costs of migration. Furthermore, our model shows that a market in inequality can exist even if the leaders do not provide an explicit benefit.

In this paper, we have attempted to explore in more depth what actually makes a region more or less circumscribed, as this has not always been clear from previous work in this area. To do this, we conceptualized the landscape as habitable patches of land and examined how connections between them affected the evolution of inequality. Although these scenarios are somewhat abstract they have allowed us to separate out two components of circumscription that can be deduced from geographical data that may be useful in assessing circumscription in real world data: connectivity and size of an area. In our model, area (or total number of polities) appears to have a stronger effect on inequality, while a high connectivity is important when the number of polities is high enough. In terrestrial environments these habitable patches are surrounded by other patches of land that are not habitable or easy to travel over, and we have explored some network structures that resemble real-world geographical landscapes. These results support the general thrust of circumscription theory as it shows that inequality does tend to be higher in the kinds of environments where there are limits to dispersal such as valleys, islands, or rivers that flood to produce fertile land in environments of otherwise low productivity. Future work could examine network structures derived from real world data. A particularly interesting application could be to look at transport networks [39] and assess how the role of circumscription has changed with time as constraints on migration move from being dictated by geography to being influenced more by the transport infrastructure in place.

A number of simplifying assumptions have been made to keep this initial model tractable. There are several ways to build on this model to make it more realistic or address related issues. First, we considered that group size is constant and that polities do not differ in their productivity. However, demographic changes will affect the degree to which an area is circumscribed, and will affect the costs and benefits of dispersal [40]. For instance, an analysis in [9] shows that inequality in Ancient Egypt fluctuated with the Nile flood and variations in weather that affected the distribution of resources and people in the landscape. Acemoglu & Robinson [41] argue that population declines caused by the black death led to greater bargaining power for workers in Western Europe and thus helped to kickstart reductions in inequalities and the establishment of more inclusive political and economic institutions. Along similar lines, a previous model has explicitly considered population dynamics (but uniform productivity) and has shown that the growth of population can fill-up the best sites, creating a form of social circumscription [20,42]. Circumscription may also be described by the gradient of productivity and the patchiness of resources rather than just ‘all-or-nothing’ barriers to dispersal [6,8]. We are currently developing extensions to our model that explicitly describe polity productivity and size, and how size can change temporally with growth and migration. Another promising extension is to more explicitly describe the processes that create fluctuations in inequality. For instance, as inequality gets high, we know from historical and anthropological evidence that subordinates can fight with dominants and potentially overthrow them (as explored in reproductive skew models considering inside options and peace incentives [22,33]). Another example is that other modelling frameworks and empirical studies have shown the importance of inheritance of resources for enabling persistent, institutionalized inequality [28,43,44]. Although our model includes some element of inheritance in terms of the degree of instability in levels of inequality, it may be useful to integrate appropriability and heritability of resources and power into this framework more explicitly.

Our model examines inequality within polities and how leaders or elites can extract more or less resources from subordinates. This model is abstract enough to be applicable to a variety of scenarios but does not explicitly consider the process of how these polities form, how elites came to be in positions of power, or the interactions between polities beyond allowing migration between them. The original version of circumscription theory is a much more complex model that invokes small groups (villages) involved in conquest warfare. If one group defeats another it subsumes it and imposes tax or tribute on it, thus creating a larger, more unequal group. Including these dynamics more explicitly will be an important step for future work as it can help to ground such models more explicitly in known processes of sociopolitical evolution. In this direction, a previous model looked at the role of circumscription on inequality considering that whole groups would get displaced to a new land after conflict, rather than the population migrating between groups as in our model [8]. However, their model assumes that empty lands are always available (though of poorer qualities) and does not explicitly consider the costs of migrating caused by the geographical features of the landscape or the presence of other groups. Other progress has been made in this direction in models in [23], which considers fusion and fission of polities through conquest warfare, and Smith & Choi [45], which explicitly examines economic interactions between agents that can lead to patron–client relationships. Modelling such processes presents several challenges such as describing the decision-making of dominants or different potential strategies. Integrating models on the fission and fusion of polities [23] with coalition formation [46] and warfare decision-making [47] could be productive ways forward. We should note that predicting the effect of integrating these process in our model is not straightforward. On the one hand, unification could suppress outside opportunities for subordinates and allow dominants to create increasingly high levels of despotism. On the other hand, larger groups could be more unstable, and lead to lower inequality. Incorporating models of the evolution of institutions that enable larger collections of groups to be stable could thus also prove valuable in this regard [48–51].

The aim of our modelling approach in this paper has been to develop a framework that connected some of the verbal reasoning presented in anthropology and archaeology with more formal models derived from evolutionary ecology. Our results show that the underlying logic of aspects of circumscription theory has some support in relation to factors that affect costs of migration away from potential despots. Our modelling process has also sought to clarify how we can conceptualize circumscription, and the consequences of considering that the outside options of subordinates can include migrating to other groups. We have not attempted to create a complete model of circumscription theory and we have outlined some of the ways in which it could be extended in future. It is also important to emphasize that the models described do not capture all the different types of hypotheses that have been proposed in the literature (see [52,53]). Circumscription theory and reproductive skew belong to a general class of ‘coercive’ hypotheses that focus only on the benefits of hierarchy or inequality to the dominant individuals or elites. There are also hypotheses that emphasize the potential group-level benefits that leaders or elites might bring despite the costs of inequality, such as improved coordination [18,54] and cooperation [19,27] of collective actions. We are currently developing models that attempt to examine a wider variety of such processes in order to assess how different processes relate to each other. Understanding the extent to which environmental circumscription may have shaped the evolution of inequality and complex societies in the real world is an empirical issue that requires collection of suitable data and appropriate analyses. As several different process may be at play in any given scenario we have to be careful to understand what predictions different hypotheses make and assess the relative importance of these different potential explanations. Models such as those developed in this paper can help sharpen our thinking about how the distribution of resources and groups within a landscape can impact the evolution of economic and socio-political relationships. In doing so it can help make predictions clearer or enhance our understanding on what factors are important to properly evaluate our hypotheses.

## Data Availability

The code is available online from the Github repository: https://github.com/CedricPerret in the project ‘TerCirNet’. The data are provided in electronic supplementary material [55].
